# Sensorimotor Grounding of Musical Embodiment and the Role of Prediction: A Review

**DOI:** 10.3389/fpsyg.2016.00308

**Published:** 2016-03-04

**Authors:** Pieter-Jan Maes

**Affiliations:** Department of Art, Music, and Theatre Sciences, IPEM, Ghent UniversityBelgium

**Keywords:** embodied music cognition, music perception, music performance, dynamical systems, predictive coding

## Abstract

In a previous article, we reviewed empirical evidence demonstrating action-based effects on music perception to substantiate the musical embodiment thesis (Maes et al., 2014). Evidence was largely based on studies demonstrating that music perception automatically engages motor processes, or that body states/movements influence music perception. Here, we argue that more rigorous evidence is needed before any decisive conclusion in favor of a “radical” musical embodiment thesis can be posited. In the current article, we provide a focused review of recent research to collect further evidence for the “radical” embodiment thesis that music perception is a dynamic process firmly rooted in the natural disposition of sounds and the human auditory and motor system. Though, we emphasize that, on top of these natural dispositions, long-term processes operate, rooted in repeated sensorimotor experiences and leading to learning, prediction, and error minimization. This approach sheds new light on the development of musical repertoires, and may refine our understanding of action-based effects on music perception as discussed in our previous article (Maes et al., 2014). Additionally, we discuss two of our recent empirical studies demonstrating that music performance relies on similar principles of sensorimotor dynamics and predictive processing.

## 1. Introduction

Under the impetus of ecological and embodied approaches to music, it is now commonly agreed that bodily states and processes and perceptual-motor interactions are inherent to music cognition (Shove and Repp, 1995; Godøy, 2003; Reybrouck, 2005; Leman, 2007; Maes et al., 2014; Schiavio et al., 2015). It is noteworthy that lately, the embodied approach becomes progressively extended from mere perception and cognition, to related areas of musical affect and emotion (Cochrane, 2010; Krueger, 2013), reward and motivation (Chanda and Levitin, 2013; Zatorre and Salimpoor, 2013), and social interaction (Moran, 2014; D'Ausilio et al., 2015). However, despite serious advances in the field, improvements could be made in order to further develop a “radical” embodiment thesis to music cognition and interaction, and maybe more important, to substantiate this thesis with empirical evidence (Mahon and Caramazza, 2008; Chemero, 2011; Wilson and Golonka, 2013; Kiverstein and Miller, 2015). The crux of the radical embodied cognition thesis is the view that music cognition emerges from the real-time interaction of modality-specific processes serving perceptual or sensorimotor functions in the brain, bodily states and dynamics, and environment. In our previous review article, we collected studies demonstrating that music perception automatically engages multi-sensory and motor processes, as well as studies demonstrating that planned or executed body movement may influence music perception (Maes et al., 2014). This evidence is typically cited in favor of the embodiment thesis given above (Maes et al., 2014; Matyja, 2015; Schiavio et al., 2015). However, as reliably argued by Mahon and Caramazza (2008), this conclusion cannot be fully drawn based on this evidence. The aim of this article is to collect further empirical evidence and computational models to substantiate the role of interaction in the embodiment thesis in the domain of music perception and performance. In a first part, we argue that (psycho)acoustic properties of sound, combined with processes within the human auditory and motor system fundamentally shape perception. On this basis, we propose an explanation of commonalities in musical repertoires across cultures, specifically related to tonality and tempo. Importantly, we will emphasize the necessity to consider the role of long-term knowledge formation through learning, and of related prediction and error-correction processes. More specifically, we consider acoustic properties, the human body, and the auditory system as natural dispositions on top of which learning and predictive processes may operate, leading to a differentiation in perception and musical repertoire. In a second part of this article, a similar approach was applied to music performance, in particular focusing on musical timing. We highlight two of our recent studies demonstrating the role of body dynamics and sensorimotor processes for timing in music performance. Again, we pinpoint how these processes may serve as a basis for learning processes facilitating prediction mechanisms.

In our approach to music perception and performance, we argue that insights from **dynamical system** theories may prove particularly useful. Previously, dynamical systems theories have been applied to the study of motor control (Turvey, 1990; Kelso, 1995; Thelen and Smith, 1998; Warren, 2006), and cognition (Port and Van Gelder, 1995; Van Gelder, 1998; Beer, 2000; McClelland et al., 2010; Buhrmann et al., 2013; Shapiro, 2013). This approach considers the various functions (sensory, motor, affective, cognitive, and social) engaged in people's interaction with music as intrinsically (non-linearly) interwoven and reciprocally deterministic. It involves a process-based approach focusing on the processes and constraints that propagate order and organization within all of the complexities, variability, and change inherent to humans' interaction with music (Ashby, 1962; Fischer and Bidell, 2007; Deacon, 2012). For that purpose, special emphasis will be given to (associative) learning, prediction and error-correction processes that underly music perception and performance, and link to core concepts of music research such as motivation, reward, affect, and agency.

KEY CONCEPT 1 **Dynamical system**

A dynamical system can be defined by some key features:

[1] Assembly: A dynamical system is composed of multiple homogeneous and/or heterogeneous components.

[2] Self-organization: A dynamical system is a self-organizing system, in which order emerges out of the interactions of its components, without being explicitly controlled from within or without.

[3] Stability: Self-organization of a dynamical system is attracted toward stable relationships between its components (cf. error minimization).

[4] Constraint- and processes-based: Interactions, self-organization, and stability within a dynamical system are tied to the disposition of its components and the processes in which they are enmeshed, in interaction with the constraints and opportunities that the environment imposes/offers.

## 2. Music perception

### 2.1. Dynamics and natural dispositions

#### 2.1.1. Pitch processing, tonal perception, and music syntactic processing

Tonality refers to a musical system of tones (i.e., pitch classes) standing in a hierarchical relationship (Lerdahl, 2001). Tonality is characteristic of musical cultures worldwide (Western, Indian, Chinese, and Arabic), throughout history (Gill and Purves, 2009). The perceived stability of particular tones and chords (i.e., their tonal function) depends on the overarching musical context in which they occur. This context dependency has commonly been interpreted as evidence for the existence of abstract cognitive music-syntactic processing, rooted in learning processes, and thus musical enculturation (Krumhansl, 1990; Tillmann et al., 2000; Janata et al., 2002). Long-term exposure to the “statistics” of tonal relationships in tonal music lead to the development of cognitive schemata that capture tonal hierarchies, leading to specific expectancies of harmonic and melodic progress. It is plausible that the statistics of occurring tonal relationships in the musical repertoire drives the development of predictive models of tonal perception. However, the predictive model does not answer the crucial question *why* the co-occurrences got into the repertoire. In the following, some key studies are presented indicating that the perception of tonal hierarchy may be grounded in dynamical, sensory-based mechanisms that draw upon inherent physical and biological constraints (i.e., acoustics, and the human auditory system).

In articles by Bigand et al. (2014) and Collins et al. (2014), an important corpus of empirical studies on tonal perception was readdressed. These studies involve a series of behavioral studies on tonal perception applying the typical harmonic priming paradigm, instructing participants to judge whether a target chord was in-tune or out-of-tune with respect to a single prime chord or a longer harmonic context. Based on the reaction time of people's responses, tone profiles reflecting tonal hierarchies could be constructed. In addition, Bigand et al. (2014) readdressed a series of event-related potential (ERP) studies showing that violations of harmonic regularities are reflected in late positive components and early negative components. Bigand et al. (2014) proposed a sensory-based model to explain the observed data. The computational model used was the auditory short-term memory (ASTM) model introduced by Leman (2000). This ASTM model was used to simulate the afore-mentioned series of empirical studies in the field of tonal music perception. The obtained simulated ratings indicated that most of the behavioral and neurophysiological responses observed in these studies could be accounted for by the ASTM model. Considering the “nature” of this auditory short-time memory, neuroimaging studies have pinpointed the role of a dynamic interplay between attentional and sensory systems (Kaiser, 2015), as well as sensorimotor processes in support of the temporary maintenance of auditory information in working memory (Schulze and Koelsch, 2012). In another study, by Collins et al. (2014), it was further argued that low-level sensory properties of musical sounds shape tonal hierarchy and expectation. Though, they concluded that musical syntax requires multiple representational stages along a sensory-to-cognition continuum. These results suggest that music-syntactic processing is, to a certain extent, a stimulus-driven process, rooted in the (psycho)acoustic properties of sound. Although different tonal systems and tuning systems are employed in different musical traditions, it is highly peculiar however that the most widely used scales typically consist of five or seven pitch classes. Additionally, research has shown that these scales have specific spectral characteristics, in the sense that the component intervals of these scales have greatest spectral similarity to a harmonic series (Gill and Purves, 2009). In the following, some studies are discussed that provide a further explanation for why these scales are preferred above others.

In a series of works, Sethares (1993, 2005) explained how the use of tuning systems and tone scales stems from the specific timbre of musical instruments being used. The timbre of tones produced by Western musical instruments is predominantly characterized by a fundamental frequency with a harmonic series of partials. Drawing on previous work by Plomp and Levelt (1965), Sethares calculated the combined dissonance among all partials of a tone, leading to a dissonance curve in which minima (i.e., local consonance) correspond with many Just Intonation scale steps. Although, not entirely undisputed (cf. Cousineau et al., 2012), sensory dissonance seems to be a property of the human auditory system related to the critical bandwidth of the basilar membrane. Hence, the use of certain scales across different cultures seems to be directly related to both the timbre of the musical instruments and the working of the human auditory system.

In another series of studies, Large and colleagues provide a neurophysiological explanation of *why* certain tone scales are preferred above others (Large, 2011a,b; Large and Almonte, 2012; Lerud et al., 2014). For that purpose, they introduced a computer model (i.e., gradient-frequency neural networks; GFNNs) of auditory pitch processing based on a non-linear, dynamical systems approach. Characteristic for the model is that the response frequencies of neural oscillators at different stages along the auditory pathway do not simply reflect the frequencies contained in the input stimulus; oscillations may occur at harmonics (e.g., 2:1 and 3:1), subharmonics (e.g., 1:2, 1:3), and more complex integer ratios (e.g., 3:2) of the stimulus frequency, and in the case of multi-frequency stimulation, at summation and difference frequencies (cf. Lee et al., 2009). According to Large and colleagues, this non-linear resonance behavior predicts the generalized preference for simple integer ratios, forming a biological basis of the most widely used tonal systems and tuning methods. Interestingly, it is further argued that internal connectivity between naturally coupled neural oscillators may be strengthened by Hebbian learning processes, providing an explanation why different tonal systems exist among different musical traditions (cf. Chandrasekaran et al., 2013). In their work, Bidelman et al. (Bidelman, 2013; Bidelman and Grall, 2014), give further evidence for a neurobiological predisposition for consonant pitch relationships, basic to tonal hierarchies. Their research indicated that perceptual correlates of pitch hierarchy are automatically and pre-attentively mapped onto activation patterns within the subcortical auditory nervous system. Their work provides further evidence that the structural foundations of musical pitch perception inherently relate to innate processing mechanisms within the human auditory system.

This corpus of research suggests that the dynamics and (short-time) processes within the auditory apparatus play a fundamental role in music perception. In addition, it is shown that the acoustic environment (e.g., timbre of musical instruments) provides the fundament of most well-known tuning systems and tone scales. This focus on auditory and environmental conditions is valuable as it may explain well *why* specific tonal relationships got into the musical repertoire. This supports our central claim that the auditory apparatus and acoustic environment provide the genuine predispositions on top of which long-term learning processes may operate to differentiate further the musical repertoire. In that regard, we agree with Koelsch stating that “long-term knowledge about the relations of tones and chords is not a necessary condition for the establishment of a tonal hierarchy, but a sufficient condition for the modulation of such establishment (that is, the establishment of such a hierarchy is shaped by cultural experience)” (Koelsch, 2012, p.105).

#### 2.1.2. Processing of time and the resonance model of tempo perception

Next to tonality, another musical feature is characteristic of the repertoire of Western music, namely tempo. van Noorden and Moelants (1999) and Moelants (2002) analyzed the perceived tempo distribution of a huge sample of Western music. The results showed that the perceived tempi in Western music group around the 500 ms period (120 bpm or 2 Hz). Interestingly, studies in the domain of motor and auditory-motor control report related findings. For instance, when people are asked to make (unconstrained) cyclical movements with a finger or wrist at a comfortable regular tempo, it is shown that their preferred tempi peak slightly below 120 bpm (Kay et al., 1987; Collyer et al., 1994). Also, research indicated that the preferred step frequency in free-cadence walking during short and extended periods is about 2 Hz (120 steps per min; Murray et al., 1964; MacDougall and Moore, 2005). Additionally, it has been shown that auditory-motor synchronization is most accurate when the tempo of the external auditory stimulus is around 120 bpm (Styns et al., 2007; Leman et al., 2013). The observed correspondence between periods of cyclic (auditory-)motor behavior and preferred musical tempi suggests a link between the musical repertoire, music perception, and the human motor system. This idea is at the center of van Noorden's and Moelants' resonance model of time perception, which ties time perception in music to natural resonance frequencies in the human body. The model views the human body, as it is capable to perform periodic movements, as a harmonic oscillator with a distinct natural resonance frequency. Resonance is the phenomenon whereby an external periodic force drives the amplitude of oscillations to a relative maximum. Resonance occurs when the frequency of the driving force is close to the natural frequency of the oscillator. In the model of van Noorden and Moelants, the external periodic force is the musical beat that drives corresponding periodic movement responses. The model suggests that the distribution of preferred tempi in the Western repertoire is matched to the natural predisposition of the human motor system to move comfortably and spontaneously at a pulse rate around 2 Hz. Again, similar to our discussion of tonality, this work strongly suggests that the “statistics” of the musical repertoire, and the preference and perception of tempo (i.e., whether a musical piece is slow or fast) are rooted in physical and biological dispositions inherent to the sensory and motor systems.

### 2.2. Predictive processing

Musical input is often noisy or ambiguous making it difficult to reliably discern basic auditory features such as pitch, duration, and dynamics. In cognitive science, it becomes common to refer to Bayesian statistical inference to explain perception under such “uncertain” conditions. The basic idea is that people constantly make predictions of ensuing sensory events, based on current sensory input and learned sensorimotor regularities in our environment. Based on Bayesian principles, these predictions may lead to more accurate (optimal) perceptions under noisy and ambiguous conditions.

This predictive framework may further refine our understanding of the action-based effects that were reviewed in our previous article, in particular disambiguation effects (Maes et al., 2014). We have explained how repeated experience of auditory-motor regularities lead to internal models of our interaction with the world. As explained, the forward component of these models allows predicting the auditory states or phenomena that go along with performed body movements. Hence, we hypothesize here that action-induced expectancies may promote a selective response to incoming auditory information during music listening causing people to “sample” the auditory features in an “optimal” way so that expectations are confirmed, and prediction errors avoided (Clark, 2015). In other words, auditory and musical features that confirm “prior beliefs” are given priority and enter into perception, leading to the observed disambiguation effects. This explanation considers perception as a process of active engagement, in which the interaction between auditory stimulation, sensorimotor predictions, and attention play a central role (Schröger et al., 2015).

In addition to action-based disambiguation effects on music perception, predictive processing based on Bayesian inference has been applied to general perception phenomena (Temperley, 2007), and more specifically to auditory scene analysis (Winkler and Schröger, 2015; note also early inferentialist approaches by Bregman, 2008 and Levitin, 2006, rhythm, Sadakata et al., 2006; Lange, 2013; Vuust and Witek, 2014, and duration Cicchini et al., 2012; Aagten-Murphy et al., 2014).

Although the concept of the “**Bayesian brain**” is promising, we should be aware that many issues are left to be resolved (Eberhardt and Danks, 2011; Jones and Love, 2011; Bowers and Davis, 2012; Marcus and Davis, 2013; Orlandi, 2014b). Two of the most prominent questions relate to how the “hypothesis space” is restricted, and to whether the Bayesian brain deploys explicit internal representations of the rules that underlie prediction. At the present, typical Bayesian inferential accounts do not offer a solution to these questions. Recent accounts may offer further clarification by approaching Bayesian perception from an embedded perspective rooted in natural scene statistics and ecology (Orlandi, 2014a,b; Judge, 2015). The debate between inferentialism and ecology is outside the scope if this article. What is important however is to acknowledge that human prediction in both accounts is closely linked to our sensitivity to statistical regularities in our environment. Then the question remains why certain regularities and rules got into the musical repertoire at the expense of others? Therefore, we reviewed empirical evidence in the previous section showing that the dynamics and natural dispositions of our acoustic environment, human body, and auditory system constrains specific regularities. Then, on top of that, cultural- and history-specific habits and preferences may further differentiate the musical repertoires.

KEY CONCEPT 2 **The Bayesian brain**

In its essence, the theory of the Bayesian brain posits that the human brain is compelled to infer the probable causes of its sensations, and to predict future states of the world. This ability relies on the existence of learned (generative/internal) models of the world, which include prior knowledge about sensorimotor regularities (cf. the concept of internal models discussed in Maes et al., 2014). This idea can be traced back to von Helmholtz' understanding of perception as inference (von Helmholtz, 1962). Nowadays, this idea is reflected in the theory of *predictive coding*, and the related *Bayesian coding hypothesis*, which stand out as most dominant accounts in psychology and neuroscience to explain the brain's functions, ranging from sensory perception to high-level cognition (Rao and Ballard, 1999; Knill and Pouget, 2004; Friston and Kiebel, 2009; Clark, 2013; Pouget et al., 2013; Summerfield and de Lange, 2014). In that regard, perception is a process of probabilistic inference, whereby perception is viewed as the “compromise” between sensations and prior knowledge, made in an attempt to minimize the discrepancy between both (cf. *free energy principle*, Friston, 2010). This can be realized in various ways: by optimizing the reliability and precision of sensory input through attention processes (Rao, 2005; Feldman and Friston, 2010; Brown et al., 2011; Kaya and Elhilali, 2014), by introducing perceptual bias (Geisler and Kersten, 2002), or by updating one's prior beliefs.

## 3. Music performance

In this section, we present two recent empirical studies in support of the central idea of this article, namely that—similar to music perception—music performance may heavily rely on the inherent dynamics of the human motor system, in combination with predictive processing allowing online adaptation to changing environments. Thereby, we focus on temporal control of performers' body movements. For musicians, it is important to temporally coordinate muscle activity in order to control their musical instrument, or vocal chords in the case of singers. Traditional accounts posit the existence of a cognitively controlled internal clock mechanism to keep track of time, indicating when muscles need to be activated (Church, 1984; Allman et al., 2014). However, as human cognitive resources are fairly limited, this account is probably incomplete to fully explain temporal behavior. Accumulating evidence is gathered demonstrating that the human sensorimotor system may support time perception and production, in addition to cognitively-based mechanisms (Hopson, 2003; Mauk and Buonomano, 2004; Ross and Balasubramaniam, 2014). In a series of experiments, we defined and tested two hypotheses to provide more insights into the factors and mechanisms regulating sensorimotor timing strategies in music performance; limited to solo performance, and regular tone production tasks. First, based on theories on **emergent timing**, it was hypothesized that the dynamic control of body movements might lead to temporal regularities with a minimum of explicit cognitive control. Second, it was hypothesized that actions might be aligned to perceived temporal patterns in self-generated auditory feedback, leading to corresponding regular motor patterns.

KEY CONCEPT 3 **Event timing vs. emergent timing**

Research on motor control and coordination suggests that the timing of rhythmic body movements is a hybrid phenomenon. Typically a distinction is made between discrete and continuous (quasi-periodic) movements (Robertson et al., 1999; Zelaznik et al., 2002; Delignières et al., 2004; Larue, 2005; Zelaznik et al., 2008; Torre and Balasubramaniam, 2009; Studenka et al., 2012; Janzen et al., 2014). Whereas discrete rhythmic movements are characterized by salient events separated by pauses in bodily movement, continuous rhythmic movements are smooth without interspersed pauses. Importantly, research suggests that these movement types rely on different control mechanisms. Discrete movements are regulated by an “event-based timing system.” Here, the basic idea is that timing is explicitly controlled by a dedicated clock capable of keeping track of time. One of the most influential accounts of event-based timing is the pacemaker-accumulator model (Gibbon, 1977). In contrast, continuous movements are regulated by an “emergent timing system,” which pertains to a dynamical systems perspective on motor control. According to this perspective, coordinated (regular) body movements are to a high extent the result of the motor system's dynamics with a minimum of explicit, central control (Turvey, 1977; Thelen, 1991; Kelso, 1995; Warren, 2006).

**Figure 1:**
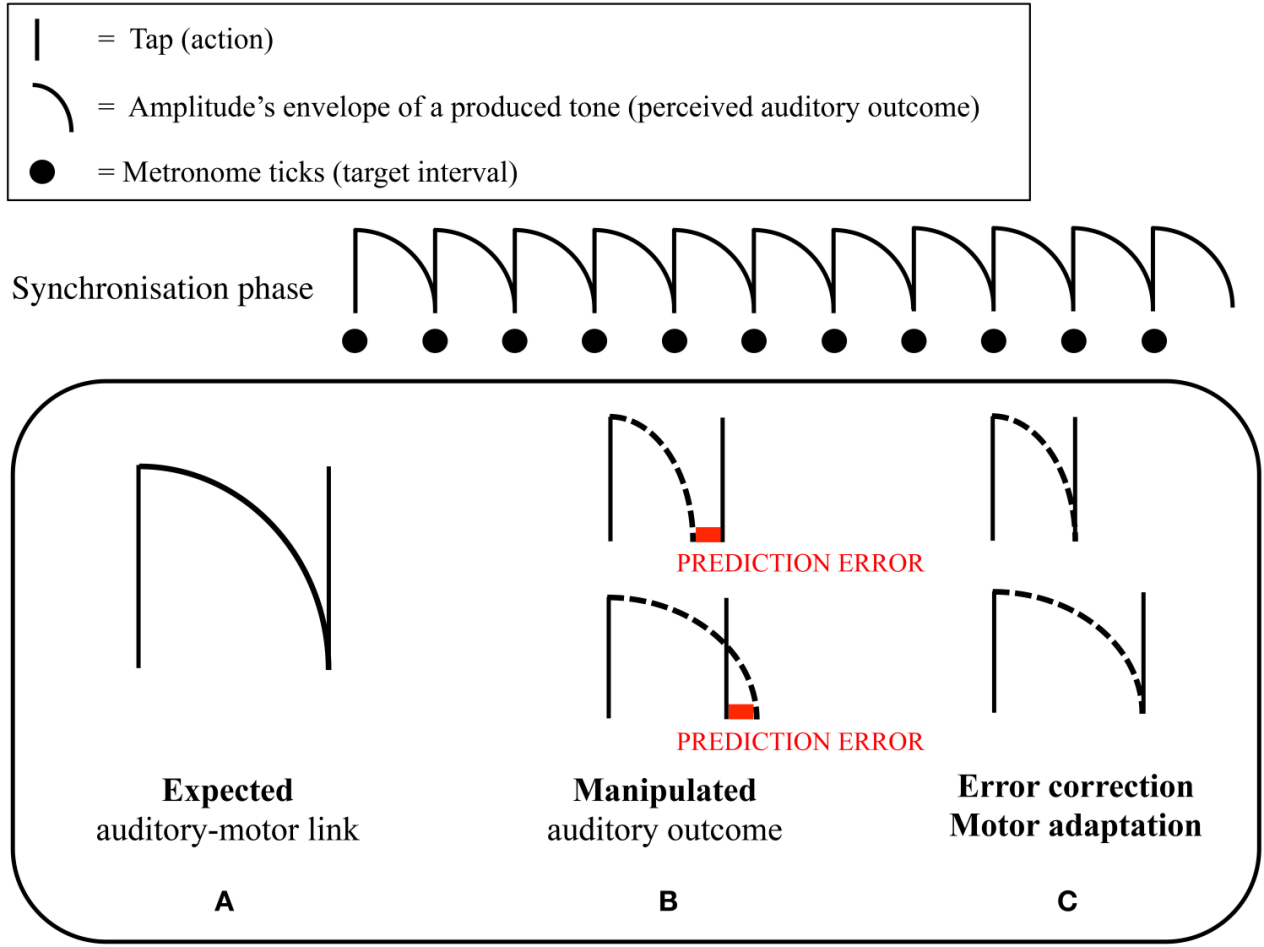
**Graphical representation of the mechanism of motor adaptation in Maes et al. (2015a)**. Participants were asked to perform a synchronization-continuation task. **(A)** Representation of the relationship between the target interval between two taps, and the amplitude's envelope of the tone that was produced by a tap. As can be seen, a tone nicely fitted the target interval. In the synchronization phase of the continuation paradigm, people learned to integrate the (fixed) tones' duration (perception), with the target interval that needed to be tapped (action) through repeated experience. **(B)** We hypothesized that participants could use the tone's amplitude's envelope as a reference to time their tapping; namely, one tapped at the moment that the previous tone ceased (i.e., sensorimotor timing strategy). Throughout the continuation phase, we gradually lengthened or shortened the duration of the produced tones. **(C)** We hypothesized that, if participants relied on a sensorimotor strategy, they would adapt their tapping pace when the tones' duration became longer or shorter throughout the continuation phase. This because of the discrepancy that appeared between the ceasing of the tone, and the time a tap was produced. Correspondingly, we expected that participants would change their tapping pace in order to avoid this discrepancy to occur. Maes et al. (2015a) found that a gradual shortening of the tones' duration resulted in an increased tapping pace. A gradual lengthening did not yield any significant effect in tapping pace.

In the experiments, we asked participants—musical novices (Maes et al., 2015a) and professional cellists (Maes et al., 2015b)—to perform melodies consisting of equally spaced notes at a specific target tempo (synchronization-continuation task) while performing an additional cognitive task (cf. dual-task interference paradigm). The main idea was that the production of regular intervals would be relatively unharmed by an additional cognitive load when participants applied a sensorimotor-based timing strategy. The results of these experiments showed that when continuous arm movements could be applied in between tone onsets, production of regular intervals in the continuation task was not affected by an additional cognitive task, suggesting the use of a sensorimotor timing strategy. This in contrast to conditions where no movement was allowed in between tone onsets, leading to a significant increase in variability of produced temporal intervals, suggesting the use of a cognitively-controlled timing strategy. Additionally, participants were generally less accurate—i.e., further apart from the target tempo—when no arm movements were allowed. In this context, Maes et al. (2015a) investigated the role of self-generated auditory feedback. It was found that participants—in particular when no additional load was present—were better able to keep the target tempo in the continuation phase when key taps produced tones that filled the complete duration of the interval, compared to when tones were short. In another part of the experiment, long tones filling the complete duration of the interval were made gradually shorter or longer throughout the continuation phase. Interestingly, it was found that, when tones were made shorter, participants speeded up their tapping tempo accordingly (i.e., intervals between produced onsets became gradually shorter; see Figure 1).

These results indicate that timing in music performance may capitalize directly on the control of movement dynamics and coupled action-perception processes, without the need to explicitly (cognitively) compute time. In other words, our findings showed that musical goals, here regular interval production, may be outsourced to the human sensorimotor system in interaction with (auditory) information accessible in the environment (Clark and Chalmers, 1998; Tylén and McGraw, 2014). Given the inherent limitations of cognitive resources, this concept of *outsourcing*—which shows resemblance with the concept of *auditory scaffolding/latching* (DeNora, 2000; Conway et al., 2009)—enables humans to optimally perform specific tasks in specific contexts, depending on their specific capabilities, state, and intentions. Nonetheless we argue that in order to be effective, sensorimotor timing strategies need to necessarily interact with processes involving associative learning, **prediction**, and **error-correction**. Basically, in synchronizing actions and tones to an external auditory metronome, associative learning facilitates to integrate movement dynamics and dynamic change in auditory information with the temporal intervals to be produced, leading to the development of so-called **internal models**. These enable then to keep the tempo in absence of the external metronome. Characteristic of people is to make predictions about the sensory consequences of planned actions. For instance in the context of our study (Maes et al., 2015a) people expected the onset of a tone to coalesce with the ending of the previous tone. Now, due to the dynamics and uncertainty inherent to internal and external conditions, discrepancies may occur between the expected sensory outcome of actions and the actual outcome. Typical for humans is to correct for occurring discrepancies by spontaneously adapting one's actions or perception, potentially leading to an update of the internal model. In Maes et al. (2015a), this was evidenced by the observation that people made temporal intervals shorter in response to a gradual shortening of the tones they produced in the continuation phase. This finding illustrates a fundamental mechanism and powerful strategy to adapt and guide temporal behavior toward specific goals. On top of that, it may evenly contribute to practical applications in the field of sports and motor rehabilitation, where strategies to adapt people's movement behavior instantaneously—and potentially unconsciously—are of high relevance (Moens and Leman, 2015).

KEY CONCEPT 4 **Internal model, prediction, error-correction, and motor adaptation**

The temporal integration of actions and their sensory outcome—acquired through systematically repeated sensorimotor experiences—establishes what is typically referred to as an *internal model* (Maes et al., 2014). Internal models contain an *inverse* and *forward* component. Forward models allow predicting the likely sensory outcome of a planned or executed action. A distinct property of forward models is that they allow transforming discrepancies between the *expected* and the *actual* sensory outcome of a performed action into an error signal, which drives changes in motor output (i.e., motor adaptation) in order to reduce sensory prediction errors (Jordan and Rumelhart, 1992; Wolpert et al., 1995; Friston et al., 2006; Lalazar and Vaadia, 2008; Norwich, 2010; Shadmehr et al., 2010; van der Steen and Keller, 2013).

## 4. Discussion

So far, the most frequently cited empirical evidence for the embodiment thesis has been grounded in the observation that music perception automatically engages multi-sensory and motor simulation processes (Schiavio et al., 2015), or that bodily states and movement may influence music perception (Maes et al., 2014). In this article, we advocated for more rigorous evidence to substantiate the “radical” embodiment thesis in the domain of music perception and performance (Mahon and Caramazza, 2008; Chemero, 2011; Wilson and Golonka, 2013; Kiverstein and Miller, 2015). Therefore, we provided a focused review presenting empirical evidence and computer models demonstrating that music perception and performance may be directly determined by the acoustics of sound and by the natural disposition and dynamics of the human sensory and motor system. On top of that, we have emphasized the role of long-term processes involving learning and prediction in how humans interact with music. At the present, the exact nature of these processes is still a matter of ongoing debate—boldly between inferential and ecological accounts (Orlandi, 2012, 2014a)—yet to be fully determined. However, the collected findings suggest to consider short-term modality-specific processes serving perceptual or sensorimotor functions, and long-term learning and prediction processes as reciprocally determined and interacting; sensorimotor experience may lead to predictions, and predictions may shape sensorimotor engagement with our environment.

In the future, it would be of interest to further extent this sensorimotor-prediction loop with aspects that relate to music expression, emotion, motivation, and social interaction. For instance, the work of Bowling et al. (2010, 2012) indicate that the musical expression and perception of happiness and sadness has a biological basis in speech. They found that the acoustic frequency spectra of major and minor tone collections, linked to, respectively, happy and sad music, correspond to the frequency spectra found in, respectively, excited and subdued speech. This finding is of particular interest as it provides an explanation why the perception of musical emotion is shared across cultures (Fritz et al., 2009). Further, it is of interest to link musical expression and the experience of affect to neurodynamical processes (Seth, 2013; Flaig and Large, 2014). In addition to musical expression, the study of the sensorimotor-prediction loop can provide deeper insights into aspects of motivation and reward in music. Previous research demonstrated that dopaminergic activity, which relate to feelings of reward, encodes learning prediction errors (Waelti et al., 2001; Schultz, 2007; Hazy et al., 2010). This link of prediction processes to actual physiological responses, in this case dopamine responses, may contribute to our understanding of feelings of pleasure and reward that arise in music-based interactions (Chanda and Levitin, 2013; Zatorre and Salimpoor, 2013). Finally, all of the processes that occur within an individual in interaction with its sensory environment, may be dynamically linked to its social environment, leading to phenomena such as interpersonal coordination and synchronization (Repp and Su, 2013; Moran, 2014; D'Ausilio et al., 2015).

It is important to note that a dynamical, process-based approach to how humans interact with music requires a severe reconsideration of currently dominating analytical tools and methods (behavioral and neurophysiological) that are often reductionistic and focus on generalizations at the expense of variation and change. Therefore, it would be of interest to systematically incorporate methods from within the field of dynamical structure analysis to take into account time-dependent changes, variability, and non-linear complexities (Amelynck et al., 2014; Badino et al., 2014; Demos et al., 2014; Teixeira et al., 2015). A dynamical, processed-based approach, together with appropriate analytical tools may contribute profoundly to our understanding of music, and more importantly, to how and why people interact with music. In turn, this knowledge may be capitalized on by more practical research in various domains, such as sports (Karageorghis and Priest, 2012), motor rehabilitation (Altenmüller et al., 2009; Särkämö and Soto, 2012), developmental disorders (Koelsch, 2009), and well-being (MacDonald et al., 2012).

## Author contributions

The author confirms being the sole contributor of this work and approved it for publication.

### Conflict of interest statement

The authors declare that the research was conducted in the absence of any commercial or financial relationships that could be construed as a potential conflict of interest.
